# Validation of a Chromosome 14 Risk Haplotype for Idiopathic Epilepsy in the Belgian Shepherd Dog Found to Be Associated with an Insertion in the *RAPGEF5* Gene

**DOI:** 10.3390/genes13071124

**Published:** 2022-06-23

**Authors:** Janelle M. Belanger, Tiina Heinonen, Thomas R. Famula, Paul J. J. Mandigers, Peter A. Leegwater, Marjo K. Hytönen, Hannes Lohi, Anita M. Oberbauer

**Affiliations:** 1 Department of Animal Science, University of California, Davis, CA 95616, USA; jmbelanger@ucdavis.edu (J.M.B.); trfamula@ucdavis.edu (T.R.F.); 2 Department of Medical and Clinical Genetics, University of Helsinki, 00014 Helsinki, Finland; tiina.heinonen@helsinki.fi (T.H.); marjo.hytonen@helsinki.fi (M.K.H.); hannes.lohi@helsinki.fi (H.L.); 3 Department of Veterinary Biosciences, University of Helsinki, 00014 Helsinki, Finland; 4 Folkhälsan Research Center, 00290 Helsinki, Finland; 5 Department of Clinical Sciences, Utrecht University, Yalelaan 108, 3584 CM Utrecht, The Netherlands; p.j.j.mandigers@uu.nl (P.J.J.M.); p.a.j.leegwater@uu.nl (P.A.L.)

**Keywords:** dog, idiopathic epilepsy, *RAPGEF5*, risk haplotype

## Abstract

An idiopathic epilepsy (IE) risk haplotype on canine chromosome (CFA) 14 has been reported to interact with the CFA37 common risk haplotype in the Belgian shepherd (BS). Additional IE cases and control dogs were genotyped for the risk haplotypes to validate these previous findings. In the new cohort, the interaction between the two regions significantly elevated IE risk. When the haplotypes were analyzed individually, particular haplotypes on both CFA14 (ACTG) and 37 (GG) were associated with elevated IE risk, though only the CFA37 AA was significantly associated (*p* < 0.003) with reduced risk in the new cohort. However, the CFA14 ACTG risk was statistically significant when the new and previous cohort data were combined. The frequency of the ACTG haplotype was four-fold higher in BS dogs than in other breeds. Whole genome sequence analysis revealed that a 3-base pair predicted disruptive insertion in the *RAPGEF5* gene, which is adjacent to the CFA14 risk haplotype. *RAPGEF5* is involved in the Wnt-β-catenin signaling pathway that is crucial for normal brain function. Although this risk variant does not fully predict the likelihood of a BS developing IE, the association with a variant in a candidate gene may provide insight into the genetic control of canine IE.

## 1. Introduction

A previous genome-wide study of idiopathic epilepsy (IE) in Belgian shepherd (BS) dogs identified two interacting haplotypes associated with IE risk: one on canine chromosome (CFA14 and one on CFA37 [1]. The relatively high prevalence of IE in the BS breed [2,3,4,5] makes this a fortuitous breed for genetic studies of canine IE. The finding of significant associations of genomic regions with IE is promising for potentially revealing causal variants. The ACTG haplotype of the CFA14 single nucleotide polymorphisms (SNPs) BICF2S23230472, BICF2P437468, BICF2S23211419, and BICF2S23539344 was associated with increased IE risk, whereas the haplotype CTCT was associated with reduced IE risk. The CFA14 risk haplotype appeared to interact with a region in close linkage disequilibrium with A Disintegrin And Metalloproteinase Domain 23 (*ADAM23*) gene, a common IE risk gene on CFA37 [4], specifically with the Kruppel-like factor (*KLF7*) gene intronic SNPs BICF2P271491 and TIGRP2P419463_rs8724220. The present study sought to confirm these findings with a larger cohort of BS dogs that met both the control and IE criteria, the latter being defined as Tier 1 using the classification of the International Veterinary Epilepsy Task Force [6]. Additionally, because the CFA14 risk haplotype was seen to coordinate risk in combination with the SNPS in full linkage disequilibrium with the *ADAM23* gene, and *ADAM23* is associated with IE in many different dog breeds [4], we asked whether the CFA14 risk haplotype is similarly prevalent across dog breeds. Thus, we determined the prevalence of the IE risk haplotype both within the BS breed and in other breeds. Furthermore, to better understand the basis of the risk haplotype, we asked whether the CFA14 risk haplotype was associated with a potentially causal variant in a candidate gene by exploring the genomic sequence in the risk haplotype region [6]. We hypothesized that because the *ADAM23* risk genotype is found in multiple dog breeds [4], the ACTG risk haplotype on CFA14 that interacts with the *ADAM23* region may also be similarly widespread in the dog population, and that the region would be associated with a functional variant in a gene related to neurological function.

## 2. Materials and Methods

To validate and build upon the original study [1], genomic DNA was collected from an additional 121 BS dogs in Europe and North America, categorized as IE cases (*n* = 47 of which 11 were from Finland, 13 from the Netherlands, and 23 from the USA), and healthy controls over the age of 10 years with no history of neurological or immune disease (*n* = 74 of which 12 were from Finland, 44 from the Netherlands, and 18 from the USA). Buccal swabs or whole blood samples were collected by owners or veterinarians, respectively, and submitted to the study. Sample DNA from the US, Finland, and the Netherlands was processed as previously described [7,8,9]. The DNA was then genotyped for the CFA14 and 37 risk SNPs using variant-specific PCR primers and a standard PCR protocol (Table S1). The amplicon sizes were verified by running 5 µL of the PCR product on a 1% agarose gel, with the remaining product purified using ExoSAP-IT™ (Thermo Fisher Scientific, MA, USA) and Sanger sequenced with an ABI 3700 Genetic Analyzer (Applied Biosystems, Foster City, CA, USA). Genotypes were assessed for contribution to IE risk using odds ratios (OR) and logistic regression, as described [1]. The ACTG haplotype frequencies were calculated in a large cohort of Belgian shepherds (*n* = 596 of which 296 were from Finland, 106 from the Netherlands, and 194 from the USA) that had been collected by the authors and genotyped using the Illumina CanineHD BeadChip (San Diego, CA, USA) with 173,662 SNPs based on the CanFam3.1 reference genome. To determine whether the risk ACTG haplotype was observed in other dog breeds, we used publicly available genomic array data [10,11,12,13,14,15,16] for breeds other than the Belgian shepherd.

DNA from seven US Belgian Tervuren were submitted to Novogene for whole genome sequencing (Illumina NovaSeq6000 Novogene, Sacramento, CA, USA) having an average coverage of 12×. Whole genome data from three IE dogs having the ACTG high-risk haplotype on CFA14 and four control dogs homozygous for the low-risk CTCT haplotype were explored for potential causal variants in the risk haplotype region. A whole genome analysis pipeline for quality checks, trimming, and mapping of raw reads was performed, as previously described [17]. The FreeBayes [18] haplotype-based caller was then used to call variants across all samples. Variant files were annotated with SnpEff version 5.0e [19] using the CanFam3.1 reference genome and Ensembl annotation version 104 [20].

## 3. Results

### 3.1. Haplotype Risk and Frequency 

**Figure 1:**
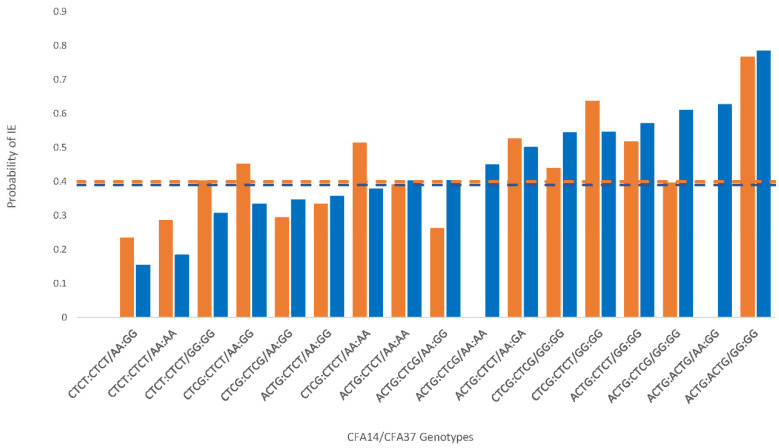
Canine chromosome 14 and 37 haplotype interactions and the probability of idiopathic epilepsy in the Belgian shepherd (BS) as a function of specific genotypic interactions. The orange bars represent the data from only the new cohort in the present study (*n* = 121), while the blue bars reflect the combined data for the new cohort and the published [1] cohort of BS dogs (*n* = 183). The dashed lines indicate the computed threshold (0.40 for orange and 0.39 for blue), with probabilities in excess of the lines indicating a significantly increased risk of IE.

The new BS cohort demonstrated a pattern similar to the original study findings [1], although at a more modest level (Table 1, Figure 1). Specifically, the CFA14 ACTG haplotype OR indicated a predisposition to IE risk, and the CTCT haplotype was associated with reduced risk but did not reach significance in the new cohort. The haplotype on CFA37 (AA) associated with lowered risk of IE was significantly lower in the new cohort of BS (*p* < 0.003). As was shown in a previous publication [1], although additional haplotype combinations were observed that differed between the original cohort of BS dogs and the new cohort assembled here, the risk haplotypes on CFA14 (ACTG) and CFA37 (GG) significantly interacted with and amplified the IE risk in the new cohort (Figure 1). When the data were combined with the published cohort data, the presence of the ACTG haplotype on CFA14 doubled the OR of IE (*p* < 0.001), and the presence of the CTCT haplotype on CFA14 was associated with a one-half lower risk of IE (*p* < 0.02).

The CFA37 *ADAM23* risk association to IE is found in multiple breeds [4] leading us to determine the ACTG risk haplotype frequency in other breeds. When the CFA14 risk haplotype frequency was evaluated in BS dogs and other breeds, the ACTG risk haplotype was four-fold more abundant in the BS, whereas the CTCT haplotype associated with lowered IE risk was twice as abundant in the BS (Table 2).

### 3.2. Candidate Gene Investigation 

The regions immediately adjacent to the SNPs on CFA14 that comprise the ACTG haplotype and those adjacent to the CFA37 haplotype were explored in the whole genome sequence data, as previously described [17]. The ACTG risk haplotype falls 17,855 bases upstream of the Rap Guanine Nucleotide Exchange Factor 5 (*RAPGEF5*) gene, which has a role during neurogenesis [21] and alterations in expression are associated with seizures such as temporal lobe epilepsy [22,23]. Whole genome sequence analysis of *RAPGEF5* (NCBI accession XM_005628616.4) revealed a 3-base pair disruptive insertion in exon 1 of *RAPGEF5*, c.87delT > GGCG (CFA14: 36208900), and p.Ala29dup, found in all IE cases having the ACTG risk haplotype and absent in the control dogs with the CTCT non-risk haplotype. Sanger sequencing of Belgian shepherd dogs (*n* = 180) showed 100% concordance between the *RAPGEF5* insertion and dogs having the ACTG risk haplotype; none of the dogs carrying the CTCG risk haplotype or the CTCT haplotype on CFA14 had insertion (Figures S1 and S2). Using publicly available whole genome sequencing data [12] for other breeds (*n* = 139), a proportion of dogs of those other breeds (7%) showed discordance between the presence of the ACTG haplotype and the *RAPGEF5* insertion (Table S2). For CFA37, the genomic sequence revealed a missense variant in exon 12 of *ADAM23* that had already been reported [8,24] and thus was not explored further. The genomic sequence of the *KLF7* gene on CFA37 was also explored, and no variants were found, further corroborating published findings [24]. 

## 4. Discussion

In a replication study, the previously identified risk haplotypes [1] trended to the same increased risk for IE in a new cohort of BS dogs, although the individual ORs failed to reach significance. Importantly, however, the previously observed interaction between the CFA14 and 37 haplotypes to increase the risk of IE was seen in the new cohort of BS dogs. Furthermore, when all the dogs from the new and the past cohort were analyzed together, the IE risk was elevated for dogs that had the ACTG haplotype on CFA14. As expected [4], the association of risk with CFA37 was also detected. In contrast to the more common risk loci on CFA37, the risk haplotype on CFA14 appears to be much more prevalent in the BS dog than in other breeds, which also corresponds to the relatively high prevalence of IE in the breed [2,3,4,5] suggesting this may be a breed-specific variant. 

The CFA14 genomic sequence in the BS was explored in the region of the ACTG risk haplotype, and a sequence variant in *RAPGEF5* was found to be in full concordance with the risk haplotype. *RAPGEF5* appears to be a good candidate gene for involvement in IE due to its role in neurological function [21,25]. The Wnt-β-catenin signaling pathway is critical for normal brain development and function [26] and a recent review on the pathogenesis of epilepsy suggests that the Wnt-β-catenin pathway could be considered a new target for treatment in epilepsy [27]. *RAPGEF5* regulates a defining step in the Wnt signaling pathway: the nuclear translocation of β-catenin [28,29]. In development, *RAPGEF5* is continually expressed [29] and in an animal model of induced epilepsy, *RAPGEF5* is downregulated within areas generating fast ripples, which have been defined as an “electrophysiological signature that precedes the onset of epilepsy” [23]. Another member of the RAPGEF family, *RAPGEF2*, has been tangentially associated with familial epilepsy in humans [30] and directly in mice with *RAPGEF2* knocked out display brain malformations consistent with those seen in human patients with epilepsy [31]. *RAPGEF2* and *RAPGEF5* share a significant amino acid identity. The present study is the first to associate *RAPGEF5* with naturally occurring IE. 

The 3-base pair INDEL in the *RAPGEF5* gene observed with the ACTG CFA14 risk variant aligns to a region immediately adjacent to the DEP domain (Dishevelled, Egl-10, and Pleckstrin domain) of the RAPGEF5 protein (Figure S3). Membrane association is critical for the function of RAPGEF5, and the DEP domain in the N-terminal region of the RAPGEF5 protein is responsible for coordinating spatial localization by directing the protein to cellular membranes, thereby facilitating interactions with other binding partners [32]. The identified insertion of an additional alanine associated with the IE risk has the potential to alter the DEP functionality by altering its 3-dimensional protein configuration [33]. Disruption of the RAPGEF5 protein function could contribute to the onset of IE in the Belgian shepherd, consistent with the increased risk observed in dogs with the CFA14 ACTG risk haplotype. Thus, our findings that a haplotype on CFA14 that doubles the relative risk for IE is associated with a potentially disruptive INDEL in *RAPGEF5* may provide insight into the regulation of IE in the BS breed.

## Figures and Tables

**Table 1:** Odds ratio (OR) and 95% confidence interval (CI) for haplotype blocks on canine chromosome 14 (CFA14) and CFA37 for Belgian shepherds for the new and combined cohorts. Haplotypes significantly associated with the risk for idiopathic epilepsy (IE) in Belgian shepherds are indicated in bold.

New Cohort					Combined Cohort			
CFA14 Four SNP Risk Haplotype	Control (*n* = 74)	IE Case (*n* = 47)	OR (95% CI)	*p*-Value	Control (*n* = 117)	IE Case (*n* = 66)	OR (95% CI)	*p*-Value
ACTG (htz or hmz *) vs.	25	20	1.45 (0.68–3.08)	3.42 × 10^−1^	31	34	2.95 (1.56–5.56)	**8.01 × 10^−4^**
non-ACTG	49	27			86	32		
CTCG (htz or hmz) vs.	13	11	1.43 (0.58–3.54)	4.87 × 10^−1^	24	19	1.41 (0.71–2.82)	3.72 × 10^−1^
non-CTCG	61	36			81	47		
CTCT (htz or hmz) vs.	69	42	0.61 (0.17–2.23)	5.08 × 10^−1^	99	55	0.32 (0.12–0.87)	**2.33 × 10^−2^**
non-CTCT	5	5			6	11		
CFA37 two SNP risk haplotype	Control (*n* = 74)	IE Case (*n* = 47)	OR (95% CI)	*p*-value	Control (*n* = 117)	IE Case (*n* = 66)	OR (95% CI)	*p*-value
GG (htz or hmz) vs.	60	41	1.59 (0.57–4.49)	4.57 × 10^−1^	85	60	2.32 (0.89–6.04)	9.03 × 10^−2^
non-GG	14	6			20	6		
AA (htz or hmz) vs.	51	19	0.31 (0.14–0.66)	**2.54 × 10^−3^**	66	21	0.27 (0.14–0.51)	**5.57 × 10^−5^**
non-AA	23	28			39	49		

* heterozygous is abbreviated htz and homozygous is abbreviated hmz.

**Table 2:** Frequency of canine chromosome 14 (CFA14) 4-SNP haplotypes associated with idiopathic epilepsy risk in the Belgian shepherd compared to other breeds.

	Belgian Shepherds 2*n* = 1192		Other Breeds 2*n* = 4484	
CFA14 Alleles	Total	% Freq	Total	% Freq
ACCG	0	0	6	0.13
ACCT	0	0	2	0.04
ACTG	233	19.55	211	4.71
ACTT	3	0.25	89	1.98
CCCG	3	0.25	1000	22.30
CCCT	8	0.67	776	17.31
CCTG	0	0	25	0.56
CCTT	0	0	77	1.72
CTCG	159	13.34	704	15.70
CTCT	786	65.94	1585	35.35
CTTT	0	0	9	0.20
Total	1192	100	4484	100

## Data Availability

All genotype data analyzed during this study are included in the published article and its supplementary information files.

## Supplementary Materials

The following supporting information can be downloaded at: https://www.mdpi.com/article/10.3390/genes13071124/s1, Table S1. PCR amplification parameters for the *RAPGEF5* exon variant, the four-variant risk haplotype on canine chromosome (CFA)14, and the two-variant risk haplotype on CFA37. Table S2. Correspondence of the presence of exon 1 *RAPGEF5* insertion with the ACTG high-risk haplotype on CFA14 in Belgian shepherds compared to other breeds. Figure S1. Alignment of exon 1 *RAPGEF5* sequence to the CanFam3.1 reference for Belgian shepherds homozygous (hmz) for the low-risk CFA14 haplotype (CTCT) and homozygous for the high-risk haplotype (ACTG). Each row represents a specific dog’s *RAPGEF5* sequence, where the identifier indicates the CFA14 haplotype, followed by zygosity and the dog’s ID number (BT = Belgian Tervuren and BS = Belgian Sheepdog). Figure S2. Sanger sequencing of *RAPGEF5* c.87delT > GGCG (CanFam3.1). (**A**) Sanger sequence from a dog with haplotype CTCT:CTCT and wild type for the *RAPGEF5* 3-base insertion. (**B**) Sanger sequence from a dog with haplotype ACTG:CTCT and heterozygote for *RAPGEF5* 3-base insertion. (**C**) Sanger sequence from a dog with haplotype ACTG:ACTG and homozygote for *RAPGEF5* 3-base insertion. Figure S3. (**A**) Evolutionary conservation of the RAPGEF5 protein. A duplicated alanine (p.Ala29dup) is present in dogs with the *RAPGEF5* 3-base insertion. (**B**) RAPGEF5 isoform X1 protein (XP_005628673.3) showing the location of the conserved domain Dishevelled, Egl-10, and Pleckstrin (DEP). The p.Ala29dup is 74 amino acids upstream from the DEP domain.

## References

[1] Belanger J., Famula T., Gershony L., Palij M., Oberbauer A. (2020). Genome-wide association analysis of idiopathic epilepsy in the Belgian shepherd. Canine Med. Genet..

[2] Famula T., Oberbauer A., Brown K. (1997). Heritability of epileptic seizures in the Belgian tervueren. J. Small Anim. Pract..

[3] Gulløv C., Toft N., Berendt M. (2012). A longitudinal study of survival in Belgian Shepherds with genetic epilepsy. J. Vet. Intern. Med..

[4] Koskinen L.L., Seppälä E.H., Weissl J., Jokinen T.S., Viitmaa R., Hänninen R.L., Quignon P., Fischer A., André C., Lohi H. (2017). ADAM23 is a common risk gene for canine idiopathic epilepsy. BMC Genet..

[5] Oberbauer A., Grossman D., Irion D., Schaffer A., Eggleston M., Famula T. (2003). The genetics of epilepsy in the Belgian tervuren and sheepdog. J. Hered..

[6] De Risio L., Bhatti S., Muñana K., Penderis J., Stein V., Tipold A., Berendt M., Farqhuar R., Fischer A., Long S. (2015). International veterinary epilepsy task force consensus proposal: Diagnostic approach to epilepsy in dogs. BMC Vet. Res..

[7] Rincon G., Tengvall K., Belanger J.M., Lagoutte L., Medrano J.F., André C., Thomas A., Lawley C.T., Hansen M.S., Lindblad-Toh K. (2011). Comparison of buccal and blood-derived canine DNA, either native or whole genome amplified, for array-based genome-wide association studies. BMC Res. Notes.

[8] Koskinen L.L., Seppälä E.H., Belanger J.M., Arumilli M., Hakosalo O., Jokinen P., Nevalainen E.M., Viitmaa R., Jokinen T.S., Oberbauer A.M. (2015). Identification of a common risk haplotype for canine idiopathic epilepsy in the ADAM23 gene. BMC Genom..

[9] van Hagen M.A., Schipper L., Oosterveer-van der Doelen M.A., Vos-Loohuis M., Gehring R., Leegwater P.A. (2020). Analysis of polymorphisms of canine Cytochrome P 450-CYP2D15. J. Vet. Pharmacol. Ther..

[10] Dahlgren S., Ziener M.L., Lingaas F. (2016). A genome-wide association study identifies a region strongly associated with symmetrical onychomadesis on chromosome 12 in dogs. Anim. Genet..

[11] Forsberg S.K., Kierczak M., Ljungvall I., Merveille A.-C., Gouni V., Wiberg M., Lundgren Willesen J., Hanås S., Lequarre A.-S., Mejer Sørensen L. (2015). The shepherds’ tale: A genome-wide study across 9 dog breeds implicates two loci in the regulation of fructosamine serum concentration in Belgian shepherds. PLoS ONE.

[12] Jagannathan V., Drögemüller C., Leeb T., Consortium D.B.V.D., Aguirre G., André C., Bannasch D., Becker D., Davis B., Ekenstedt K. (2019). A comprehensive biomedical variant catalogue based on whole genome sequences of 582 dogs and eight wolves. Anim. Genet..

[13] Olsson M., Tengvall K., Frankowiack M., Kierczak M., Bergvall K., Axelsson E., Tintle L., Marti E., Roosje P., Leeb T. (2015). Genome-wide analyses suggest mechanisms involving early B-cell development in canine IgA deficiency. PLoS ONE.

[14] Stern J.A., Hsue W., Song K.-H., Ontiveros E.S., Luis Fuentes V., Stepien R.L. (2015). Severity of mitral valve degeneration is associated with chromosome 15 loci in whippet dogs. PLoS ONE.

[15] Vaysse A., Ratnakumar A., Derrien T., Axelsson E., Rosengren Pielberg G., Sigurdsson S., Fall T., Seppälä E.H., Hansen M.S., Lawley C.T. (2011). Identification of genomic regions associated with phenotypic variation between dog breeds using selection mapping. PLoS Genet..

[16] Wolf Z.T., Brand H.A., Shaffer J.R., Leslie E.J., Arzi B., Willet C.E., Cox T.C., McHenry T., Narayan N., Feingold E. (2015). Genome-wide association studies in dogs and humans identify ADAMTS20 as a risk variant for cleft lip and palate. PLoS Genet..

[17] Gershony L.C., Belanger J.M., Hytönen M.K., Lohi H., Oberbauer A.M. (2021). Whole Genome Sequencing Reveals Multiple Linked Genetic Variants on Canine Chromosome 12 Associated with Risk for Symmetrical Lupoid Onychodystrophy (SLO) in the Bearded Collie. Genes.

[18] Garrison E., Marth G. (2012). Haplotype-based variant detection from short-read sequencing. arXiv.

[19] Cingolani P., Platts A., Wang L.L., Coon M., Nguyen T., Wang L., Land S.J., Lu X., Ruden D.M. (2012). A program for annotating and predicting the effects of single nucleotide polymorphisms, SnpEff: SNPs in the genome of Drosophila melanogaster strain w1118; iso-2; iso-3. Fly.

[20] Howe K.L., Achuthan P., Allen J., Allen J., Alvarez-Jarreta J., Amode M.R., Armean I.M., Azov A.G., Bennett R., Bhai J. (2021). Ensembl 2021. Nucleic Acids Res..

[21] Bithell A., Alberta J., Hornby F., Stiles C.D., Williams B.P. (2003). Expression of the guanine nucleotide exchange factor, mr-gef, is regulated during the differentiation of specific subsets of telencephalic neurons. Dev. Brain Res..

[22] Knott S., Forty L., Craddock N., Thomas R.H. (2015). Epilepsy and bipolar disorder. Epilepsy Behav..

[23] Winden K.D., Bragin A., Engel J., Geschwind D.H. (2015). Molecular alterations in areas generating fast ripples in an animal model of temporal lobe epilepsy. Neurobiol. Dis..

[24] Seppälä E.H., Koskinen L.L., Gulløv C.H., Jokinen P., Karlskov-Mortensen P., Bergamasco L., Baranowska Körberg I., Cizinauskas S., Oberbauer A.M., Berendt M. (2012). Identification of a novel idiopathic epilepsy locus in Belgian Shepherd dogs. PLoS ONE.

[25] Bithell A., Hsu T., Kandanearatchi A., Landau S., Everall I.P., Tsuang M.T., Chana G., Williams B.P. (2010). Expression of the Rap1 guanine nucleotide exchange factor, MR-GEF, is altered in individuals with bipolar disorder. PLoS ONE.

[26] Barrett K.T., Choudhary A., Charkhand B., Scantlebury M.H. (2022). Animal Models in Epileptic Spasms and the Development of Novel Treatment Options. J. Clin. Neurophysiol..

[27] Yang C., Shi Y., Li X., Guan L., Li H., Lin J. (2022). Cadherins and the pathogenesis of epilepsy. Cell Biochem. Funct..

[28] Griffin J.N., Del Viso F., Duncan A.R., Robson A., Hwang W., Kulkarni S., Liu K.J., Khokha M.K. (2018). RAPGEF5 regulates nuclear translocation of β-catenin. Dev. Cell.

[29] Alharatani R., Griffin J.N., Liu K.J. (2019). Expression of the guanine nucleotide exchange factor, RAPGEF5, during mouse and human embryogenesis. Gene Expr. Patterns.

[30] Ishiura H., Mitsui J., Yoshimura J., Matsukawa M.K., Fujiyama A., Toyoshima Y., Kakita A., Takahashi H., Suzuki Y., Sugano S. (2018). Expansions of intronic TTTCA and TTTTA repeats in benign adult familial myoclonic epilepsy. Nat. Genet..

[31] Maeta K., Hattori S., Ikutomo J., Edamatsu H., Bilasy S.E., Miyakawa T., Kataoka T. (2018). Comprehensive behavioral analysis of mice deficient in Rapgef2 and Rapgef6, a subfamily of guanine nucleotide exchange factors for Rap small GTPases possessing the Ras/Rap-associating domain. Mol. Brain.

[32] Consonni S.V., Maurice M.M., Bos J.L. (2014). DEP domains: Structurally similar but functionally different. Nat. Rev. Mol. Cell Biol..

[33] Jeong W.H., Lee H., Song D.H., Eom J.-H., Kim S.C., Lee H.-S., Lee H., Lee J.-O. (2016). Connecting two proteins using a fusion alpha helix stabilized by a chemical cross linker. Nat. Commun..

