# Supplementary material for: Validation of a Chromosome 14 Risk Haplotype for Idiopathic Epilepsy in the Belgian Shepherd Dog Found to Be Associated with an Insertion in the RAPGEF5 Gene
Source: Genes (Basel). 2022 Jun 23;13(7):1124. doi: 10.3390/genes13071124 (PMC9323784; doi:10.3390/genes13071124)
Supplement: Supplementary file 1 [file genes-13-01124-s001.zip › genes-1741818-supplementary.pdf]

CFA	CanFam3.1 Location (bp)	SNP (rs ID)	SNP Description	Forward Sequence (5' -> 3')	Reverse Sequence (5' -> 3')	Product Size (bp)	Temp ˚C	Time (sec)	Cycles
14	36208900	n/a	RAPGEF5 exon 1 variant	CGTGGGCTCCGTCAAGAT	CCTCCCCAACTTCCAGCTG	298	62	35	35
14	36227069	BICF2S23230472 (rs22299660)	SNP 1 CFA 14 Haplotype	CGCCCCTTTAAACAAAATGTCC	CACCAAGCCCACAACCTATG	186	62	30	30
14	36239817	BICF2P437468 (rs22308514)	SNP 2 CFA 14 Haplotype	AAATGGCCAATGGGTATTGA	ACCAGGGTCATGAGGAGTTG	488	62	30	30
14	36258515	BICF2S23211419 (rs22331418)	SNP 3 CFA 14 Haplotype	TCTGGTGTGGTTCCTTCTCC	GCAATCCCCTTATTACCTGATCC	211	62	30	30
14	36287383	BICF2S23539344 (rs22376250)	SNP 4 CFA 14 Haplotype	ACCACTTTTACCACCCAGCT	TGTCACAGTTGGTTGGCATG	247	62	30	30
37	15527401	BICF2P271491 (rs23992883)	SNP 1 CFA 37 Haplotype	CAAAGGACTGTGGGCAAAGA	GTTCTCTCCTCTGCTACCCA	235	62	30	30
37	15597532	TIGRP2P419463 (rs8724220)	SNP 2 CFA 37 Haplotype	GCCTTTATTGTCAGTCCCACG	CCCCTTTGCCTGGTGATTTT	243	62	30	30

Table S1. PCR amplification parameters for the *RAPGEF5* exon variant, the four-variant risk haplotype on canine chromosome (CFA) 14, and the two-variant risk haplotype on CFA37.

CFA14 haplotype and RAPGEF5 3-bp insertion	Belgian shepherds (n = 180)	Other Breeds (n = 139)
ACTG with insertion	65	5
ACTG no insertion	0	1
non-ACTG with insertion	0	9**
non-ACTG no insertion	115	124

** Two dogs have ACCT with insertion

Table S2. Correspondence of the presence of the exon 1 *RAPGEF5* insertion with the ACTG high risk haplotype on CFA14 in Belgian shepherds compared to other breeds.

```
RAPGEF5 CanFam3.1      G GCC GCG GCG GCG GCG GCG GCG GCG GCG GC_ __T GAC GGC GCC CTG CGC CGC AGC CCC GGC GCC CGC GAG CAG GAG CGC GAA
CTCT hmz(BT1032)       G GCC GCG GCG GCG GCG GCG GCG GCG GCG GC_ __G GAC GGC GCC CTG CGC CGC AGC CCC GGC GCC CGC GAG CAG GAG CGC GAA
CTCT hmz(BT1241)       G GCC GCG GCG GCG GCG GCG GCG GCG GCG GC_ __G GAC GGC GCC CTG CGC CGC AGC CCC GGC GCC CGC GAG CAG GAG CGC GAA
CTCT hmz(BT1259)       G GCC GCG GCG GCG GCG GCG GCG GCG GCG GC_ __G GAC GGC GCC CTG CGC CGC AGC CCC GGC GCC CGC GAG CAG GAG CGC GAA
CTCT hmz(BT1355)       G GCC GCG GCG GCG GCG GCG GCG GCG GCG GC_ __G GAC GGC GCC CTG CGC CGC AGC CCC GGC GCC CGC GAG CAG GAG CGC GAA
ACTG hmz(BS0085)       G GCC GCG GCG GCG GCG GCG GCG GCG GCG GCG GCG GAC GGC GCC CTG CGC CGC AGC CCC GGC GCC CGC GAG CAG GAG CGC GAA
ACTG hmz(BS0549)       G GCC GCG GCG GCG GCG GCG GCG GCG GCG GCG GCG GAC GGC GCC CTG CGC CGC AGC CCC GGC GCC CGC GAG CAG GAG CGC GAA
ACTG hmz(BS1062)       G GCC GCG GCG GCG GCG GCG GCG GCG GCG GCG GCG GAC GGC GCC CTG CGC CGC AGC CCC GGC GCC CGC GAG CAG GAG CGC GAA
ACTG hmz(BS1050)       G GCC GCG GCG GCG GCG GCG GCG GCG GCR GCG GCG GAC GGC GCC CTG CGC CGC AGC CCC GGC GCC CGC GAG CAG GAG CGC GAA
```

Figure S1. Alignment of exon 1 *RAPGEF5* sequence to the CanFam3.1 reference for Belgian shepherds homozygous (hmz) for the low risk CFA14 haplotype (CTCT) and homozygous for the high-risk haplotype (ACTG). Each row represents a specific dog's *RAPGEF5* sequence where the identifier indicates the CFA14 haplotype followed by zygosity and the dog's ID number (BT = Belgian Tervuren and BS = Belgian Sheepdog).

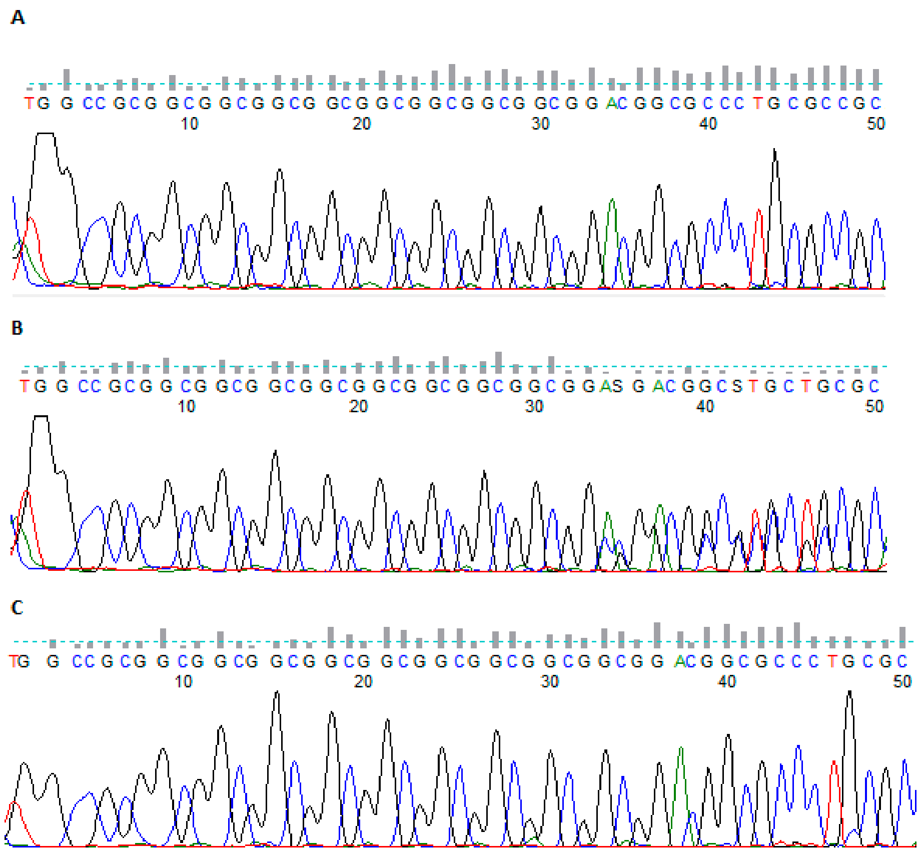

Figure S2. Sanger sequencing of RAPGEF5 c.87delT>GGCG (CanFam3.1). (A) Sanger sequence from a BS dog with haplotype CTCT:CTCT and wild type for the RAPGEF5 3-base insertion. (B) Sanger sequence from a BS dog with haplotype ACTG:CTCT and heterozygote for the RAPGEF5 3-base insertion. (C) Sanger sequence from a BS dog with haplotype ACTG:ACTG and homozygote for the RAPGEF5 3-base insertion. (FinchTV 1.4.0 Geospiza, Inc.; Seattle, WA, USA; http://www.geospiza.com archived on 01/06/2006).

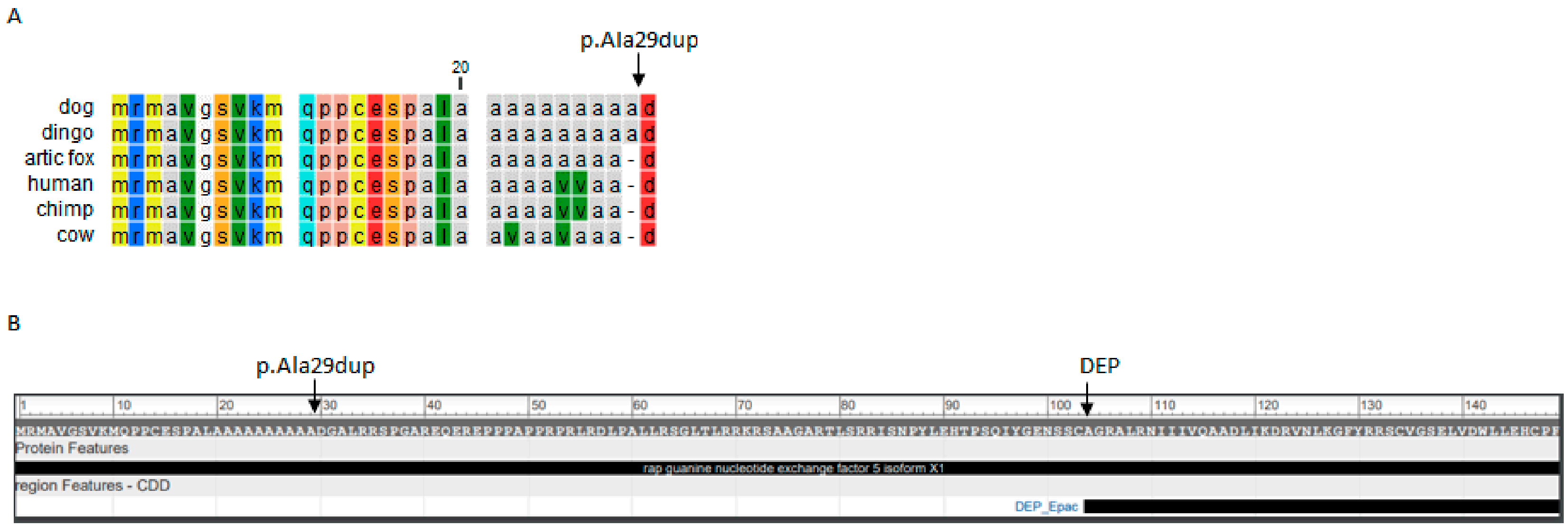

Figure S3. (A) Evolutionary conservation in the RAPGEF5 protein (CLC Genomics Workbench 22.0 QIAGEN; Redwood City, CA, USA; https://digitalinsights.qiagen.com/ accessed on 04/01/2022). A duplicated alanine (p.Ala29dup) is present in dogs having the RAPGEF5 3-base insertion. (B) RAPGEF5 isoform X1 protein (XP_005628673.3) showing the location of the conserved domain Dishevelled, Egl-10, and Pleckstrin (DEP). The p.Ala29dup is 74 amino acids upstream from the DEP domain. (Protein. National Library of Medicine (US), National Center for Biotechnology Information; 2004. Bethesda, MD, USA. Available online: https://www.ncbi.nlm.nih.gov/protein/ accessed on 04/19/2022).
